# Human cardiac organoids: multidimensional integration and clinical translation potential

**DOI:** 10.3389/fphar.2026.1859103

**Published:** 2026-07-23

**Authors:** Xiao Jin, Xinyue Ding, Zongjun Liu

**Affiliations:** Cardiology Department, Putuo Hospital, Shanghai University of Traditional Chinese Medicine, Shanghai, China

**Keywords:** cardiac organoids, clinical translation, intelligent biomanufacturing, regenerative medicine, single-cell sequencing

## Abstract

Human cardiac organoids are important *in vitro* models for cardiovascular disease research, drug screening, and regenerative medicine. Unlike traditional reviews that focus on single technological advances, this article adopts a multi-dimensional integration perspective to systematically summarize innovative breakthroughs in the construction of cardiac organoids through intelligent biomanufacturing, neuro-vascular-immune co-construction, and single-cell omics. On this basis, we analyze the unique value of the integrated human-derived three-dimensional models in revealing novel disease mechanisms, and evaluate their current application status in personalized drug toxicity prediction and cardiac regeneration and repair. Through an interdisciplinary integration perspective, this review provides a theoretical framework and practical reference for advancing cardiac organoids from basic research toward clinical practice.

## Graphical Abstract

**Figure:**
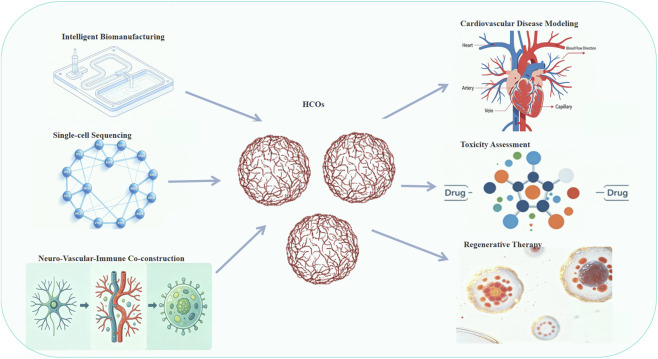

## 1 Introduction

In 2023, the World Heart Federation (WHF) released the World Heart Report. For decades, cardiovascular diseases have been the leading cause of death globally. In 2021, 20.5 million people died from cardiovascular diseases, accounting for approximately one-third of all global deaths. The number of deaths from cardiovascular diseases was 12.1 million in 1990 and 18.6 million in 2019 (World Heart Federation WHF, 2023). Therefore, exploration in the cardiovascular field is extremely urgent. In the United States, the development of a new drug to market approval takes approximately 12–15 years (Drugs, 2025), and the median research and development cost for drugs approved by the United States Food and Drug Administration (FDA) from 2009 to 2018 was 985 million US dollars (Wouters et al., 2020). Unfortunately, one-third of new drugs are still withdrawn due to clinical safety issues, with cardiovascular toxicity occurring more frequently than hepatotoxicity (Laverty et al., 2011). This not only results in significant financial losses but also introduces considerable risks into clinical trials. Therefore, developing a new model for more efficient and safer drug development in the clinical setting is more critical than ever.

Traditional two-dimensional (2D) cell culture captures valuable biological and regulatory information of cardiomyocytes and cardiac fibroblasts. However, cells grown on flat, rigid surfaces are exposed to an environment distinct from that in tissues. This affects cellular behavior, leading to insufficient maturation in terms of morphology, differentiation, and differences in cell-cell and cell-matrix interactions, which has become a major obstacle for disease modeling and the clinical translation of cell therapies (Mohr et al., 2022; Sacchetto et al., 2020; Lombardozzi et al., 2025). Animal models, on the other hand, suffer from species differences, resulting in inefficient clinical translation. Studies have shown that approximately 90% of drugs fail in early clinical studies due to excessive reliance on experimental animals (Ro, 2025; Hope and Bailey, 2025). The high failure rate in clinical trials primarily stems from safety and efficacy issues that animal data fail to predict. This also drives the average cost of bringing new medical therapies to market to between 13 and 40 million US dollars, while potentially successful treatments for humans are missed. In April 2025, the FDA proposed replacing animal testing for monoclonal antibody therapies and other drug development with more effective human-relevant methods. The FDA will promote the use of laboratory-grown human organoids and organ-on-a-chip systems to test drug safety. Laboratory testing based on human organ models and real-world human data can provide patients with faster and more reliable treatments, while also reducing research and development costs and drug prices, thereby promoting public health (FDA, 2025).

With advances in materials science and cell biology, tissue engineering has emerged as a field. In the 2010s, the rise of organoids and bioprinting significantly improved the accuracy of experimental models in simulating human cardiac physiology and pathology. By seeding cardiomyocytes derived from human pluripotent stem cells onto prefabricated biomaterial scaffolds, researchers successfully constructed engineered myocardial tissues capable of spontaneous contraction, demonstrating the feasibility of creating cardiac function *in vitro* (Lee et al., 2019; Noor et al., 2019). In 2021, Sasha Mendjan’s team at the Institute of Molecular Biotechnology in Austria achieved a milestone breakthrough. Moving away from reliance on physical scaffolds, they used precise temporal control of signaling molecules to induce human pluripotent stem cells to self-organize into an organoid with a defined lumen and rhythmic, coordinated contractions. This model, termed “Cardioids,” provided the first proof that cell clusters themselves can recapitulate early heart morphogenesis without complex external scaffolding, marking the beginning of forming specific cardiac regions through self-organization (Hofbauer et al., 2021). In 2023, the Moretti team constructed complex organoids containing an epicardial cell layer. The integration of the epicardium, a key signaling center in heart development, greatly enhanced the model’s authenticity and research potential (Meier et al., 2023). In the same year, Sasha Mendjan’s team established a human cardioid platform capable of recapitulating the developmental processes of all major embryonic heart chambers, including the left and right ventricles, atria, outflow tract, and atrioventricular canal, and for the first time observed coordinated “heartbeat” conduction across chambers, similar to that in the early fetal heart (Schmidt et al., 2023). Subsequently, multiple research teams have focused on introducing endothelial cells into human cardiac organoids (hCOs) to build internal microvascular networks. The addition of vascular cells has significantly improved the maturity and contractile strength of the organoids (Abilez et al., 2025; Voges et al., 2023). However, purely self-assembling models also face challenges, such as demanding culture conditions, significant batch-to-batch variability, and limitations in size and maturity. Purely engineered models, on the other hand, have shortcomings in biomimetic complexity. Consequently, the most cutting-edge trend is no longer an either-or choice but a creative integration of the strengths of both approaches. In recent years, hCOs constructed through interdisciplinary approaches have demonstrated significant advantages in terms of human origin, three-dimensional structure, multi-cell coordination, microenvironment controllability, and patient specificity. However, current hCOs still face challenges including insufficient functional maturation, poor long-term stability, and a lack of standardization. To more clearly elucidate these key differences, advantages, and limitations, Table 1 provides a systematic comparison of the three models.

**TABLE 1:** Key feature comparison of traditional cardiac models versus interdisciplinary hCOs.

Feature	Traditional 2D monolayer culture	Animal models	Interdisciplinary hCOs
Cell source	Usually primary cells or cell lines (animal or human)	Animals (mouse, rat, etc.), non-human	Human pluripotent stem cells (hiPSCs/hESCs); patient-specific possible
3D architecture	None; planar growth	Yes; native whole-heart structure	Yes; chamber-like structures via 3D bioprinting or self-assembly
Cellular composition and heterogeneity	Single or few cell types; lacks native cell-cell communication	Complete (cardiomyocytes, fibroblasts, endothelial, immune, neural cells)	Controlled multi-cell co-culture (vascular, immune, neural cells can be included); complexity lower than *in vivo*
Microenvironment mimicry	Very poor (rigid plastic/glass; no mechanical forces or flow)	Real *in vivo* microenvironment (hemodynamics, neurohumoral regulation)	Partial key cues mimicked via microfluidics, bioprinting (shear stress, cyclic stretch, matrix stiffness)
Functional maturity	Low (immature morphology, electrophysiology, contraction)	High (fully mature adult heart function)	Moderate (improved by vascularization and mechanical stimulation; typically fetal/neonatal-like)
Clinical predictivity/translational efficiency	Low (oversimplified model; fails to predict complex drug responses)	Low (species differences; ∼90% drug failures in clinical trials; poor cardiotoxicity prediction)	Higher (human-based; successfully models doxorubicin/trametinib cardiotoxicity and inherited heart diseases)
Throughput/standardization	High (suited for large-scale screening)	Low (costly, time-consuming, low throughput)	Moderate (standardization and automated culture being developed)
Cost and time	Low	High (housing, breeding, ethics, long duration)	Moderate (higher upfront cost, but may reduce late-stage drug failures)
Ethical concerns	Low	High (animal welfare, 3 R principles)	Low to moderate (no direct animal use; hiPSC source requires ethical oversight)
Key advantages	Simple, low-cost, high-throughput, human cells possible	Systemic complexity; real neuro-humoral integration	Human-based + 3D + multi-cell synergy + controllable microenvironment + patient specificity; models human-specific development/disease; reduces animal use; higher translational potential
Major limitations	No 3D structure or mechanical cues; distorted cell-cell/matrix interactions; functional immaturity	poor clinical prediction; costly/slow; cannot fully recapitulate human-specific disease mechanisms	Functional maturity still below adult heart; limited long-term stability; lack full autonomic and immune integration; standardization lacking; size constraints

Based on this, this review adopts “interdisciplinary integration enabling clinical translation” as its core theme. It elaborates on interdisciplinary innovations in hCO construction from three technological dimensions: intelligent biomanufacturing providing structural controllability, neuro-vascular-immune co-construction enhancing physiological fidelity, and single-cell omics resolving molecular heterogeneity. The review then analyzes the in-depth applications of hCOs built upon these technologies in cardiovascular disease mechanism research, including specific examples of both hereditary and acquired diseases. Subsequently, we summarize the current research status and value of hCOs in emerging clinical translation scenarios such as personalized drug screening, toxicity assessment, and cardiac regenerative therapy. Through this multi-dimensional integration perspective, this review aims to provide a comprehensive and cutting-edge reference for interdisciplinary research and clinical translation practices in this field.

## 2 Interdisciplinary innovation in hCOs construction technologies

### 2.1 Biomanufacturing innovations

Traditional hCOs construction relies on stem cell self-organization, which suffers from issues such as poor structural uniformity and insufficient functional maturity (Cho et al., 2022). In recent years, intelligent biomanufacturing technologies have developed rapidly. Their deep integration with hCOs construction has become a key breakthrough in addressing the aforementioned problems and represents the innovative core distinguishing it from traditional construction methods. With 3D bioprinting and microfluidic chip technology at its core, intelligent biomanufacturing enables precise regulation, achieving the accurate replication of hCO’s “structure-function-microenvironment”.

Previous research has shown that mixing already differentiated cardiomyocytes, fibroblasts, and other cells with biomaterials (such as hydrogels) and forming them via 3D bioprinting can create heart models that closely simulate pumping function (Kim M. et al., 2025). However, such models often prioritized functional output over structural complexity, resulting in relatively uniform tissues. To address this limitation and achieve greater biomimicry, advances in printing technologies such as photopolymerization-based and inkjet bioprinting have been crucial (Groll et al., 2018; Moroni et al., 2018). Consequently, 3D bioprinting technology is no longer confined to constructing structurally uniform engineered heart tissues. Its higher resolution and gentler processing conditions are now being used to guide stem cell self-assembly, aiming to build hCOs with complex internal chamber structures and spatial arrangements of multiple cell types, more closely resembling the real heart development process (Kupfer et al., 2020). The Matthew team developed a 3D-printed ring-shaped scaffold composed of a hydrogel (gelatin-alginate) construct, encapsulating a mixture of human heart AC16 cardiomyocytes (CMs), fibroblasts (CFs), and microvascular endothelial cells (ECs), and used it as a Cardiac Tissue. Through rheological analysis, scanning electron microscopy (SEM), and attenuated total reflectance Fourier-transform infrared spectroscopy (ATR-FTIR), the scaffold was confirmed to possess ideal mechanical properties, a porous microstructure, and long-term stability. Flow cytometry showed that the viability of the 3 cell types significantly improved and continued to proliferate over the 21-day culture period. Close heterogeneous cell coupling and interactions among the different cardiac cell types were observed, along with the exhibition of contractile function during sustained culture. These results collectively indicate that this 3D bioprinted “hCOs” model can be used to simulate the cardiac environment and is applicable for drug cytotoxicity screening or investigating potential disease mechanisms (Alonzo et al., 2022).

Microfluidic chip technology provides organoids with a precise microenvironment regulation platform. The “organ-on-a-chip” system based on microfluidic chips can accurately regulate the flow rate of culture medium, oxygen concentration, and nutrient gradients, simulating the hemodynamic microenvironment within the body (Shao et al., 2026). One study developed a microfluidic platform with a composite structure: the lower layer consisted of a methacrylated gelatin (GelMA) microgroove (MG) chip loaded with cardiomyocytes, while the upper layer was a polydimethylsiloxane (PDMS) support structure. The PDMS layer not only served for encapsulation but also featured two internally designed side chambers that provided pathways for cyclic mechanical actuation and medium perfusion to the cardiomyocytes. These two side chambers were connected to the main microfluidic platform via microchannels. Upon activation of the computer control program, a vacuum pump initiated operation, applying periodic negative pressure to the platform’s tubing system. This negative pressure was transmitted to the chip’s side chambers, inducing regular deformation of the flexible PDMS walls. Consequently, cyclic mechanical stretch stimuli mimicking cardiac pulsations were applied to the cardiomyocytes cultured within the chambers. This established a system that promotes cardiomyocyte maturation through periodic mechanical actuation, significantly influencing cell phenotype, contraction patterns, and calcium transients (Wang et al., 2025). In addition, cardiomyocytes, endothelial cells, and cardiac fibroblasts derived from human induced pluripotent stem cells are mixed in specific proportions to form cardiac microtissue spheroids containing a primitive vascular network *in vitro*. These microtissue spheroids are then embedded into a chamber within a chip, which is filled with fibrin hydrogel pre-mixed with additional vascular cells. Inside the chip, the self-generated vascular network within the microtissue automatically connects with the externally growing vascular network in the hydrogel, leading to the formation of a complete, perfusable vascularized system within approximately 7 days. This transforms existing three-dimensional heart models into more physiologically relevant organ models (Arslan et al., 2024). In summary, the use of perfusable microfluidic systems can significantly enhance the physiological fidelity of hCOs and their application potential in drug screening and disease modeling.

### 2.2 Innovations in multicellular platforms

The natural heart is a complex organ composed of cardiomyocytes, nerve cells, vascular endothelial cells, immune cells and other cells. The synergy between cells is the key to maintaining the normal function of the heart (Litviňuková et al., 2020). Traditional hCOs construction focuses on the differentiation and maturity of cardiomyocytes, neglecting the synergy of nerve, blood vessel and immune system, resulting in organoid unable to completely simulate the physiological and pathological state of the heart *in vivo* (Calvillo et al., 2019). In recent years, “multi-cell collaborative construction” has become an innovative direction of hCOs research, which significantly improves the physiological simulation of organoids by integrating neural, vascular and immune cell systems.

Vascularization construction is a core step in enhancing the survival and functional maturation of hCOs. In 2017, a study simulated the natural process of intramyocardial coronary vasculogenesis in the embryonic heart to construct vascularized hCOs. This was achieved by three-dimensional co-culture of human induced pluripotent stem cell-derived cardiomyocytes with specifically selected human endothelial cells and mesenchymal cells that support vessel formation *in vitro*, using defined cell types and ratios. These cell mixtures were able to self-organize into functional myocardial tissue containing lumenized vascular networks without the need for scaffolds, laying an important foundation for hCOs vascularization (Richards et al., 2017). In 2021, after transplanting hCOs with cardiovascular features into mice, their cardiac function was maintained through an *in vivo* vascularization process. This demonstrated that the model could establish connections with the host circulatory system in a living environment, providing a basis for its future application in cardiac regenerative medicine and more in-depth disease modeling research (Lee et al., 2022). Furthermore, vascularized hCOs are crucial for simulating the key pathological feature of abnormal extracellular matrix (ECM) deposition around cardiomyocytes and vascular cells during cardiac fibrosis (Tian et al., 2022). In 2022, the Liang team successfully induced stem cells to simultaneously differentiate into beating cardiomyocytes and cardiac endothelial-like cells by dynamically regulating Wnt signaling, forming chamber-like structures enveloped by cardiac endothelial cells. This achieved the integrated development of myocardial and vascular precursor cells. Compared to organoids composed solely of cardiomyocytes, these chambered organoids containing vascular endothelial cells exhibited more mature action potentials and displayed more sensitive and pronounced pathological responses to toxic drugs. This indicates that the introduction of vascular cells and the formation of chamber structures collectively enhance the functional maturity of the organoids and the reliability of disease modeling, providing a superior model platform for cardiovascular development research and drug screening (Liang et al., 2022).

Based on the successful construction of vascularized hCOs, an increasing number of studies are exploring the related mechanisms. In 2023, a research team revealed how vascular cells enhance the functional maturity and disease modeling capability of hCOs through specific intercellular communication mechanisms. Vascular cells upregulate ECM deposition via paracrine platelet-derived growth factor receptor beta (PDGFRβ) signaling, thereby increasing the overall contractile force of the organoids. Furthermore, in disease modeling, researchers confirmed that vascular cells determine the severity of diastolic dysfunction induced by inflammatory factors and identified the key paracrine role played by endothelin-1 (ET-1) in this process (Voges et al., 2023). This finding cannot be recapitulated in monolayer co-culture. The interaction between the ECM and integrins activates the ROCK-LIMK-Cofilin phosphorylation signaling pathway. The activation of this pathway further induces the initiation of the TGFβ/NODAL and PDGF pathways, which collectively promote cardiomyocyte maturation and vascular network formation (Noh et al., 2023). The Wen team demonstrated that circSARS-CV2-N1368 enhances platelet adhesion to endothelial cells, inhibits endothelial vasodilation, and suppresses exogenous cell proliferation and migration, thereby reducing angiogenesis and the beating of hCOs. Uptake of miR-103a-3p can reverse the circSARS-CV2-N1368-induced endothelial injury. Evidence that SARS-CoV-2-related circRNA impairs endothelial function and suppresses organoid beating can likewise only be captured in human-derived three-dimensional models (Wen et al., 2025).

Distinct from the mature construction of vascularized organoids, enhancing the representation of neural cell populations within organoids remains a significant challenge. In 2024, Andrew’s team replaced the commercial Matrigel matrix with N-cadherin peptide-functionalized gelatin methacryloyl hydrogel (GelMA-Cad). This GelMA-Cad material provides directed biochemical signals through the bioactive peptides immobilized within the matrix to guide specific cell fate decisions and promote functional maturation, steering the fate selection of neural stem cells. This approach offers valuable insights for the neuralization of hCOs (Kjar et al., 2024). Recent research has established a comprehensive method for constructing functional neuro-cardiac junction (sympathetic innervation) organoid models. This study not only achieved, for the first time, the generation of organoids mimicking peripheral sympathetic ganglia from human pluripotent stem cells (hPSCs), but more crucially, it fused these ganglion organoids with hCOs to create an assembloid platform capable of studying the interactions between the two. Immunofluorescence staining confirmed the invasive growth of neuronal axons into the cardiac tissue. Utilizing electrophysiological techniques such as calcium imaging and patch-clamp, the study directly verified that electrical stimulation of sympathetic neurons or application of norepinephrine could specifically accelerate the beating frequency of the hCOs, an effect reversible by beta-blockers, thereby proving the successful establishment of functional innervation. The long-term benefits of this model are significant. It not only provides the first *in vitro* simulation of human sympathetic ganglion development and its interaction with target organs, offering a unique human model platform to study the mechanisms of neuromodulation dysregulation in diseases like heart failure and arrhythmias, but also holds promise for application in high-throughput drug screening to discover novel therapeutic strategies targeting the “neuro-cardiac” axis. Furthermore, it opens new pathways for research in autonomic nervous system developmental biology (Liu et al., 2026).

The integration of immune cells enables hCOs to simulate inflammatory microenvironments. Traditional organoids, lacking immune cells, fail to replicate the immune regulatory mechanisms involved in disease processes. In 2025, a highly complex 3D hCOs model derived from human iPSCs was introduced. This model spontaneously organizes under three-dimensional culture conditions, developing functional myocardium with spontaneous rhythmic contractions while simultaneously forming a branched network of endothelial-lined vascular lacunae, endothelial cords, and capillary-like structures. The key breakthrough in immune-cardiac integration for this model lies in the coordinated development of hemogenic endothelium with myocardial tissue, which subsequently differentiates to produce erythrocytes and CCR2-negative tissue-resident macrophages. These macrophages integrate into the myocardial tissue, thereby mimicking the natural co-developmental process of immune cells and cardiomyocytes during heart development. Consequently, this study constructs a complex three-dimensional platform comprising cardiomyocytes, fibroblasts, endothelial cells, and macrophages, opening entirely new avenues for investigating the specific roles of macrophages in human heart development and diseases such as myocarditis and post-heart failure repair (Rockel et al., 2025). In 2025, Aguirre’s team presented a human heart-macrophage assembloid (hHMA) model, constructing a “myocardium-vascular-immune” tripartite synergistic organoid model. By integrating embryonic monocytes derived from autologous human pluripotent stem cells (hPSCs) into hCOs, they generated physiologically relevant embryonic cardiac tissue-resident macrophages (TRMPs) that persist long-term and promote cardiogenesis. Multi-omics results indicated that these TRMPs can modulate cardiac paracrine signaling, execute cytotrophic hyperplasia, and regulate extracellular matrix remodeling and electrical conduction. In modeling the inflammatory response of atrial fibrillation, this system demonstrated immune cell infiltration and cytokine secretion profiles consistent with *in vivo* observations, providing a precise model for studying the regulatory role of immune cells in cardiac diseases and a novel platform for screening anti-inflammatory drugs (O'Hern et al., 2025).

### 2.3 Omics-driven mechanistic insights

The intricate process of building hCOs—encompassing stem cell differentiation, multicellular interactions, and dynamic microenvironmental regulation—generates immense molecular complexity. Traditional bulk analysis methods often obscure the nuanced, cell-type-specific mechanisms at play. The advent of single-cell omics technologies, particularly single-cell RNA sequencing (scRNA-seq), has revolutionized this landscape by providing an unbiased, high-resolution lens to dissect these processes. This approach has not only become an indispensable analytical tool but also a driving force for innovation in optimizing hCOs fidelity and application (Kim H. et al., 2025; Tan et al., 2024).

A primary application of scRNA-seq is to decode the molecular correlates of structural and functional maturation in hCOs. Early studies leveraged transcriptome-wide analyses to benchmark advanced models. For instance, Li et al. demonstrated that their chambered hCOs model exhibited upregulation of cardiac maturation genes compared to 2D cultures, correlating with superior physiological function (Li et al., 2018). Ho et al. further employed scRNA-seq to longitudinally track the metabolic and electrophysiological gene signature shifts during cardiomyocyte maturation within hCOs, and used this knowledge platform to establish a robust disease model of cardiac hypertrophy (Ho et al., 2022). Beyond benchmarking, scRNA-seq unveils the mechanistic impact of the cellular microenvironment. Zhang’s team revealed how a multicellular, ECM-rich niche promoted cardiomyocyte maturity and identified a specific cardiac fibroblast subtype (high in DLK1) induced by endothelial co-culture, linking this cellular crosstalk to improved therapeutic outcomes *in vivo* (Zhang et al., 2022). Similarly, Pompilio’s team used scRNA-seq to prove that culturing hCOs on a soft, embryo-like substrate enriched maturation-related gene programs, resulting in cardiomyocytes with more adult-like functional properties (Santoro et al., 2025). This precise, cell-level insight is equally transformative for disease modeling, allowing researchers to pinpoint pathogenic cell states and pathways. A comparative transcriptomic study in 2024 highlighted how different construction protocols directly shape hCOs maturity and heterogeneity, offering a blueprint for tailoring models to specific research needs (Hyams et al., 2024). More directly, Kostina et al. applied scRNA-seq to hCOs exposed to diabetic conditions, uncovering cell-type-specific stress responses in epicardial cells and cardiomyocytes, and delineating the exact lipid metabolism pathway disrupted in diabetic embryonic cardiomyopathy (Kostina et al., 2024). In immunocompetent models, Aguirre’s team utilized scRNA-seq to dissect how integrated macrophages alter cardiac cell composition and drive pro-arrhythmic signaling in a model of chronic inflammation (O'Hern et al., 2025).

Finally, single-cell omics serves as a critical tool for validating and understanding therapeutic interventions. When evaluating potential therapies, scRNA-seq moves beyond phenotypic observation to reveal mechanistic action. Cao et al. exemplified this by using scRNA-seq to demonstrate that protective extracellular vesicles (sEVs) rescued radiation-damaged hCOs by restoring metabolic and mitochondrial gene programs, providing molecular-level evidence for their therapeutic potential (Cao et al., 2024). In summary, single-cell omics has transitioned from a descriptive technology to a central, hypothesis-generating engine in hCOs research. By mapping the molecular landscape of development, disease, and treatment with unprecedented resolution, it provides the essential mechanistic insights needed to rationally engineer more accurate heart models and translate their promise into clinical understanding. Table 2 summarizes the interdisciplinary innovations in the field of hCOs construction.

**TABLE 2:** Interdisciplinary innovation in hCOs construction technologies.

Author	Year	Ref.	Evidence source/Study model	Key findings	Limitations
Kim M, et al.	2025	Kim et al. (2025a)	3D Bioprinted Engineered Cardiac Tissue	Demonstrated that 3D bioprinting a mixture of pre-differentiated cardiac cells and biomaterials can construct simple cardiac tissues that mimic pumping function	Uniform tissue architecture lacks complex chambers; limited cell maturation; long-term stability not validated
Kupfer ME, et al.	2020	Kupfer et al. (2020)	Technical Study (3D Bioprinted Chambered Organoid)	Developed a 3D bioprinted, chambered cardiac organoid that enables *in situ* expansion, differentiation, and electromechanical coupling of human cardiac muscle	Limited printing resolution; lack of vascular network; difficulties in large-scale production
Alonzo M, et al.	2022	Alonzo et al. (2022)	3D Bioprinted Ring-shaped hCO Model	Developed a 3D bioprinted ring-shaped hydrogel scaffold hCO model, confirming its ideal mechanical properties, cell viability, and sustained contractile function	Small organoid size; only 3 cell types, lacking neural and immune cells; long-term functional maintenance not evaluated
Shao H, et al.	2026	Shao et al. (2026)	Technical Review/Methodological Article	Discussed the effects of multicellularity, biochemistry, and perfusion on vessel network morphogenesis in a microfluidic vasculature-on-a-chip	Physiological relevance of vascular networks needs further validation; limited throughput for high-content applications
Wang YH, et al.	2025	Wang et al. (2025)	Microfluidic Chip System Integrating Mechanical Stimulation	Integrated melt electrospinning writing and microfluidics to engineer a cardiac microenvironment model for high-fidelity drug screening, capable of applying mechanical stimulation	Complex system, difficult to standardize; maintenance of cell phenotype during long-term culture remains challenging
Arslan U, et al.	2024	Arslan et al. (2024)	Microfluidic Chip-based Vascularized Cardiac Microtissue Model	Established a protocol for generating and characterizing hiPSC-derived, perfusable vascularized cardiac microtissues-on-a-chip	Limited maturity of vascular networks; lack of functional immune cells; still relatively low throughput
Richards DJ, et al.	2017	Richards et al. (2017)	Self-organizing Vascularized hCOs (Specific Cell Co-culture)	For the first time, constructed self-organizing vascularized hCOs containing lumenized vascular networks without scaffolds by co-culturing specific cell types	Vascular density and stability insufficient; lack of innervation; significant batch-to-batch variability
Lee SG, et al.	2022	Lee et al. (2022)	*In Vivo* Transplantation Study (Vascularized hCOs)	Demonstrated that the generated hiPSC-derived heart organoids are structurally and functionally similar to the heart, and can vascularize and maintain function upon transplantation *in vivo*	Validated only in mice, not in large animals; long-term immune rejection not assessed; limited functional integration
Tian Y, et al.	2022	Tian et al. (2022)	Disease Modeling Study (Cardiac Fibrosis)	Found that immunosuppressants Tacrolimus and Sirolimus revert the cardiac antifibrotic properties of p38-MAPK inhibition in 3D hiPSC-heart organoids	Model lacks immune cells, cannot simulate inflammation-driven fibrosis; mechanism based solely on drug intervention
Liang PY, et al.	2022	Liang et al. (2022)	Chambered Vascularized hCOs (Wnt Signaling Guidance)	Directed human pluripotent stem cells into vascularized cardiac organoids with chamber-like structures via Wnt signaling	Chamber structures do not fully mimic native heart; long-term electrophysiological stability not validated
Noh JM, et al.	2023	Noh et al. (2023)	Mechanistic Study (Signaling Pathway)	Revealed that the activation of the LIMK/Cofilin signaling pathway via ECM-integrin interactions is critical for generating mature and vascularized cardiac organoids	Relies mainly on chemical inhibitors, lacks genetic validation; crosstalk between pathways not deeply explored
Wen YH, et al.	2025	Wen et al. (2025)	Mechanistic Study (circRNA Function)	Discovered that SARS-CoV-2-derived circSARS-CV2-N1368 impairs endothelial cell function by upregulating ATF7 to activate TLR4/NF-κB/ROS signaling	Only one circRNA tested; *in vivo* relevance not fully validated; therapeutic strategies not explored
Kjar A, et al.	2024	Kjar et al. (2024)	Biomaterials Study (Functionalized Hydrogel)	Developed biofunctionalized gelatin hydrogels that support the development and maturation of iPSC-derived cortical organoids	Study focused on neural organoids; direct application to hCOs requires validation; long-term safety unknown
Liu Y, et al.	2026	Liu et al. (2026)	Neuro-Cardiac Assembloid	Modeled sympathetic ganglion development and its functional crosstalk with the heart using hPSC-derived organoid models	Innervation density lower than *in vivo*; lack of parasympathetic components; functional tests limited to acute stimulation
Rockel AF, et al.	2025	Rockel et al. (2025)	Complex Self-organizing hCOs (Containing Immune Cells)	Showed that tissue-resident macrophages co-develop with myocardial tissue in hiPSC-derived organoids	Functional diversity of macrophages not fully characterized; inflammatory response simulation limited; long-term maintenance not reported
O'Hern C, et al.	2025	O'Hern et al. (2025)	Human Heart-Macrophage Assembloid (hHMA)	Constructed human heart-macrophage assembloids to mimic immune-cardiac interactions and enable arrhythmia disease modeling	Macrophages from a single source; no neural integration; *in vitro* inflammatory environment differs greatly from *in vivo*
Li RA, et al.	2018	Li et al. (2018)	Single-cell Omics Analysis (Chambered hCOs)	Utilized transcriptomic analysis to demonstrate that their bioengineered miniature ventricular chamber model upregulates cardiac maturation genes compared to 2D cultures	Only compared to 2D, not to animal models; small sample size; functional validation insufficient
Ho BX, et al.	2022	Ho et al. (2022)	Single-cell Omics Analysis (hCOs Disease Modeling)	Robustly generated human-chambered cardiac organoids for improved cardiovascular disease modeling, utilizing data including scRNA-seq	Model mainly recapitulates hypertrophy, not other disease types; long-term stability not evaluated
Zhang F, et al.	2022	Zhang et al. (2022)	Single-cell Omics Analysis (Multilineage Cardiac Organoids)	Mapped the single-cell atlas of hiPSC-derived multilineage cardiac organoids, resolving cellular heterogeneity and interactions	Atlas static, lacking temporal dynamics; predicted cell-cell communication not functionally validated
Santoro R, et al.	2025	Santoro et al. (2025)	Single-cell Omics Analysis (Culture Condition Optimization)	Demonstrated that conditions combining geometrical confinement and substrate stiffness serve as an optimized *in vitro* model for producing cardiac organoids	Only a limited range of substrate stiffness tested; long-term functional maturity not validated
Hyams NA, et al.	2024	Hyams et al. (2024)	Single-cell Omics Analysis (Comparing Protocols)	Utilized transcriptomics to improve the design of human cardiac organoids by analyzing the effects of different protocols	No standardized protocol provided; reproducibility across different laboratories unknown
Kostina A, et al.	2024	Kostina et al. (2024)	Single-cell Omics Analysis (Disease Model: Diabetes)	In a human heart development organoid model, identified that ER stress and lipid imbalance drive diabetic embryonic cardiomyopathy	Model represents embryonic stage, not applicable to adult diabetes; lipid intervention strategies not validated *in vivo*
Cao H, et al.	2024	Cao et al. (2024)	Single-cell Omics Analysis (Therapeutic Intervention Evaluation)	Demonstrated that small extracellular vesicles from umbilical cord mesenchymal stem cells alleviate radiation-induced cardiac organoid injury by restoring metabolic programs	Radiation model not fully consistent with clinical radiotherapy; long-term safety and efficacy not evaluated

## 3 Deep application of hCO in disease mechanism research

The reason hCOs are able to uncover previously elusive pathogenic mechanisms lies fundamentally in their multi-dimensional integration strategy: the structural and mechanical controllability provided by biofabrication, the physiological relevance brought by multicellular co-culture, and the molecular precision enabled by single-cell omics. These three elements are not merely pieced together but are mutually reinforcing, allowing researchers to achieve simultaneous observation and intervention at the structural, cellular, and molecular levels within a single organoid platform. The following sections elaborate on the specific applications of integrated hCOs in both hereditary and acquired cardiovascular diseases, organized according to the integration dimensions on which each mechanism relies.

The mechanical environment of the heart is completely absent in traditional static cultures, and biomanufacturing technology can precisely recreate these conditions. When these mechanical cues are missing, certain pathological processes are significantly attenuated or fail to appear. Take myocardial ischemia-reperfusion injury as an example. Static 2D cultures or non-perfused organoids cannot simulate the shear stress changes at the moment of reperfusion, nor can they dynamically track mitochondrial fission. Using a microfluidic perfusion system, researchers can precisely administer a Drp1 inhibitor at the onset of reperfusion and monitor contractile force and mitochondrial superoxide levels in real time. They found that inhibiting excessive mitochondrial fission markedly reduces cardiomyocyte death—a protective effect that could hardly be quantified in non-perfused models (Lees et al., 2024). Similarly, radiation-induced cardiac damage exhibits a strong dependence on perfusion conditions: only in continuously perfused hCOs does radiation lead to a significant decrease in mitochondrial membrane potential and metabolic capacity, and the protective effect of mesenchymal stem cell-derived small extracellular vesicles is also manifested only under perfusion (Cao et al., 2024). This means that even when molecular pathways are identical, the absence of the mechanical environment distorts the phenotype.

However, the mechanical environment is only one part of integration. Pathological processes in the native heart are rarely accomplished by a single cell type alone; vascular endothelial cells, sympathetic neurons, and tissue-resident macrophages form complex paracrine and electrical signaling networks with cardiomyocytes. Multicellular platforms are designed to capture this network effect. In studies of cardiac fibrosis, traditional 2D cardiomyocyte monocultures can only observe endothelin-1-induced cell hypertrophy but cannot reproduce diastolic dysfunction. In contrast, vascularized hCOs (containing cardiomyocytes, endothelial cells, and fibroblasts) clearly show that endothelial cells upregulate extracellular matrix deposition via PDGFRβ signaling, and that the amount of endothelin-1 secreted directly determines the severity of inflammatory factor-induced diastolic dysfunction (Voges et al., 2023; Noh et al., 2023). Without the vascular component, the PDGFRβ-ET-1 axis would be completely missed. Studies of sympathetic innervation provide a similar example: by fusing human sympathetic ganglion organoids with hCOs and using a functionalized hydrogel to guide axon outgrowth, researchers demonstrated for the first time *in vitro* that electrical stimulation of sympathetic neurons or application of norepinephrine specifically accelerates hCO beating, and that this response can be reversed by β-blockers (Liu et al., 2026). Conventional hCOs without innervation cannot simulate this neuro-cardiac connection and therefore cannot be used for screening drugs that act on the autonomic nervous system. The incorporation of immune cells is equally indispensable. In heart-macrophage assembloids, macrophages not only promote cardiogenesis and matrix remodeling but also drive pro-arrhythmic signaling under chronic inflammation (O'Hern et al., 2025). Non-immune organoids would simplistically attribute the inflammatory response to the cardiomyocytes themselves, ignoring the amplifying role of macrophages.

The multicellular interactions described above rely on the dissection of cellular heterogeneity, which is exactly the strength of single-cell omics. Single-cell transcriptome sequencing can reveal differences in rare subpopulations and molecular features that are inaccessible to traditional bulk analysis. However, a transcriptomic map is not by itself a mechanism; causal validation still requires the cooperation of biomanufacturing and multicellular platforms. This principle is powerfully demonstrated in inherited cardiovascular disorders. For congenital heart disease, patient-specific cardiomyocytes have been shown to recapitulate disease features such as myocardial hypertrophy and contractile dysfunction, and genomic sequencing has precisely pinpointed causative mutations (e.g., TBX5, NKX2.5, GATA4) that disrupt key developmental pathways such as Notch and Wnt/β-catenin (Zhang and Wu, 2024). Take diabetic embryonic cardiomyopathy as an example. Kostina et al. (Kostina et al., 2024) performed scRNA-seq on hCOs cultured under high-glucose and high-lipid conditions and identified FADS2 as the most significantly downregulated gene in the lipid metabolism network, as well as the upstream ER stress sensor IRE1. If bulk RNA-seq had been used, the differential expression of FADS2 between cardiomyocytes and endothelial cells would have been averaged out and lost. However, proving that IRE1 indeed reduces very-long-chain fatty acids by degrading FADS2 mRNA required intervention experiments under conditions of a perfusable microfluidic system and endothelial co-culture. Similarly, after long-term culture of hCOs derived from patients with Duchenne muscular dystrophy, scRNA-seq revealed the loss of polysialic acid localization, increased ER stress, and the coexistence of multiple pathways such as hypertrophy, fibrosis, and adipogenesis (Marini et al., 2022). This cellular heterogeneity would have been masked by traditional methods. In patient-specific organoids for arrhythmogenic right ventricular cardiomyopathy and hypertrophic cardiomyopathy, single-cell omics identified SCN5A channel abnormalities and the hypercontractile phenotype caused by MYBPC3 variants, respectively, providing precise targets for subsequent drug screening (Zhu et al., 2025; Desai et al., 2024).

**FIGURE 1:**
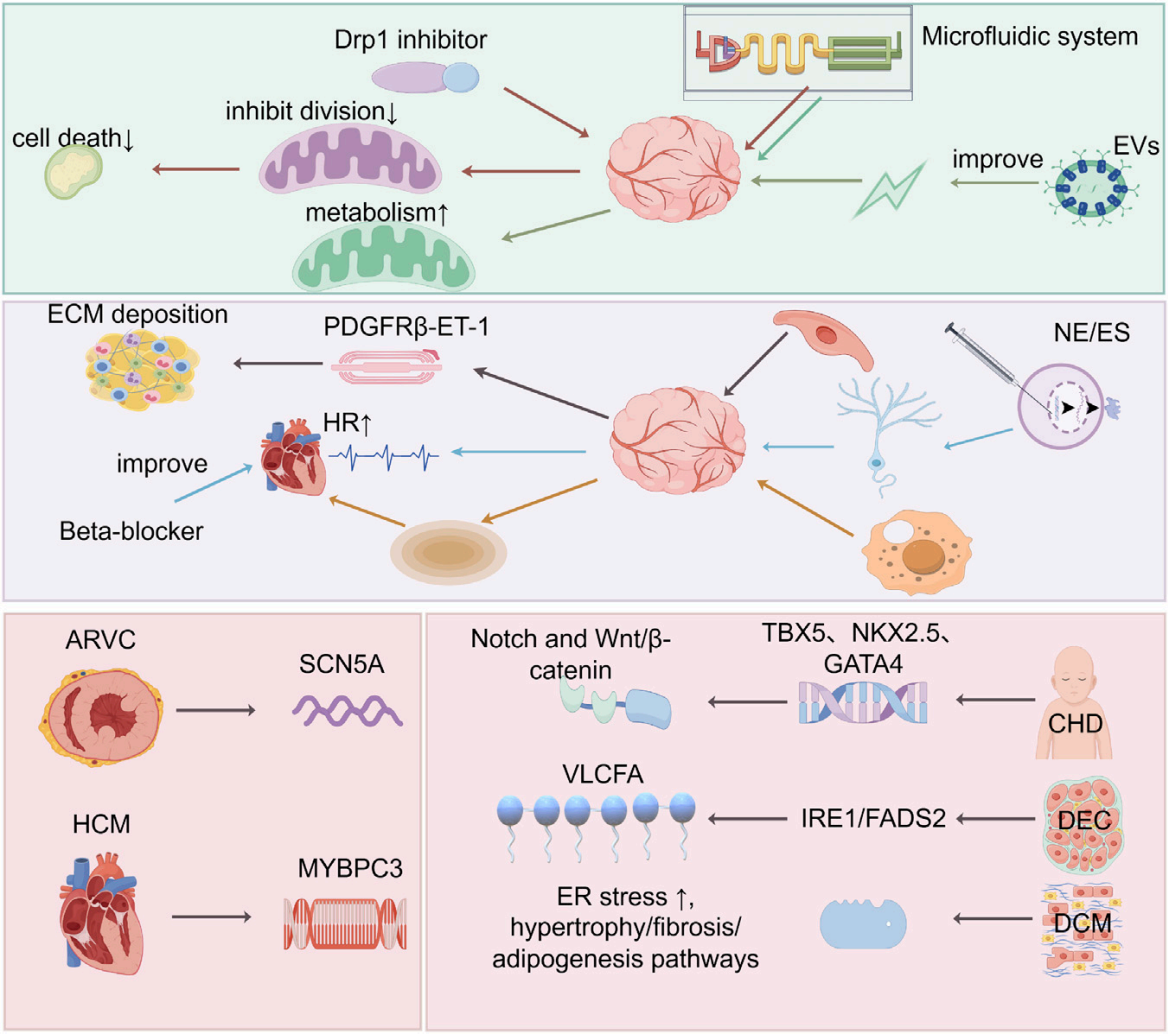
Application of hCO in disease mechanism research. Legend: Under perfusion conditions, organoids enable quantification of the anti-apoptotic effect of Drp1 inhibitors and the protective effect of mesenchymal stem cell-derived vesicles, whereas non-perfused models significantly attenuate or abolish these phenotypes. At the multicellular level, vascularized organoids demonstrate that the PDGFRβ–ET-1 axis mediates endothelial cell-dependent diastolic dysfunction; innervated organoids provide the first *in vitro* validation of sympathetic neuronal acceleration of heart rate and its reversal by β-blockers; and macrophage integration reveals their amplifying role in promoting arrhythmogenesis under chronic inflammation. hCOs derived from patient hiPSCs can faithfully recapitulate disease-specific features during long-term culture. For instance, in CHD models, mutations in genes such as TBX5, NKX2.5, and GATA4 have been identified, allowing researchers to trace the downstream disruption of key developmental pathways, such as Notch and Wnt/β-catenin signaling. Patient-specific hCOs for ARVC identified SCN5A as a critically affected sodium channel protein, and KA showed potential therapeutic efficacy by targeting this channel. Furthermore, hCOs elucidated the cellular basis of HCM in models carrying the MYBPC3 D389 V variant. hCOs from DMD patients successfully mimicked the progressive cardiomyopathy of the disease, demonstrating metabolic stress, fibrosis, and adipogenesis. hCOs: Human cardiac organoids; HR: Heart rate; CHD: Congenital Heart Disease; ARVC: Arrhythmogenic Right Ventricular Cardiomyopathy; SCN5A: Sodium voltage-gated channel alpha subunit 5; HCM: Hypertrophic Cardiomyopathy; DMD: Duchenne Muscular Dystrophy; NE:Norepinephrine; ES: Electrical Stimulation; ET-1: Endothelin-1; DCM: Diabetic Cardiomyopathy; MIRI: Myocardial Ischemia-Reperfusion Injury; EVs: Extracellular Vesicles; VLCFA: Very-Long-Chain Fatty Acid; DEC: Diabetic Embryonic Cardiomyopathy; IRE1: Inositol-requiring enzyme 1; FADS2: Fatty Acid Desaturase 2.

The above examples already demonstrate the value of pairwise integration, but some mechanisms can only be fully discovered and validated when structural controllability, multicellular synergy, and molecular resolution are present simultaneously. This three-layer evidence “structure-cell-molecule” can only be obtained simultaneously in fully integrated organoids. For example, Voges et al. (Voges et al., 2017) applied cryoinjury to hCOs and used scRNA-seq to track proliferative changes in different cell subpopulations, finding that immature organoids can completely recover function within 2 weeks, and that this regenerative response requires paracrine signals provided by endothelial cells and fibroblasts. In simple, non-perfused, non-multicellular models, such a regenerative phenotype would easily be masked by hypertrophy or fibrosis. Figure 1 summarizes the use of hCO in the study of disease mechanisms (Figure 1).

## 4 Emerging clinical transformation scenarios and values of hCOs

The above mechanistic studies not only elucidate the nature of diseases but also extract multiple potential drug targets and quantifiable functional phenotypes. Whether these findings can be translated into clinical applications depends on the availability of high-throughput, highly biomimetic testing systems. The structural controllability enabled by biomanufacturing and real-time monitoring capabilities, the organ-level cellular interactions provided by multi-cell platforms, and the molecular precision afforded by single-cell omics, these three factors collectively determine the practical value of hCOs in drug screening, toxicity prediction, and regenerative repair. The following sections elaborate on the translational applications according to the main integration dimensions on which they rely.

In drug discovery and efficacy assessment, the core contribution of biomanufacturing technology lies in providing a high-throughput platform that is both perfusable and capable of real-time readouts. Mills et al. utilized a 3D bioprinted 96-well plate hCO system to dynamically record the contractile function and calcium transients of 105 pro-regenerative compounds simultaneously. The results showed that approximately 40% of the compounds appeared safe in 2D assays but exhibited significant functional side effects under 3D perfusion conditions. This discrepancy directly stems from the presence or absence of mechanical feedback within the three-dimensional structure. If relying solely on 2D or static 3D models, the potential risks of these compounds would have been missed. Further proteomic analysis revealed that effective compounds synergistically activate the mevalonate pathway to drive cell cycle re-entry, a mechanism validated through inhibition by statins (Mills et al., 2019). Thus, biomanufacturing provides the throughput and technical feasibility for screening, while omics explains the mechanism—both are indispensable.

For cardiotoxicity evaluation, the value of multi-cell platforms is particularly prominent. Traditional 2D monocultures or non-vascularized organoids fail to capture multi-cell synergistic toxicity. Taking doxorubicin as an example, animal models poorly predict its cardiotoxicity. However, studies using vascularized, perfusable hCO platforms have revealed that endothelial cells significantly amplify cardiomyocyte damage through endothelin-1 signaling (Wu et al., 2025; Harihara et al., 2025; Jang et al., 2024; Chen et al., 2023). Similarly, the toxicity mechanism of trametinib was uncovered in vascularized hCOs as being initiated by damage to endothelial cells, which then indirectly leads to cardiomyocyte dysfunction through paracrine signaling (Beck et al., 2022); using only cardiomyocyte monoculture would directly attribute the toxicity to the drug’s attack on cardiomyocytes, thereby overlooking endothelium protection as a potential intervention strategy. Furthermore, the presence of macrophages alters the arrhythmogenic risk profile (O'Hern et al., 2025), and the developmental toxicity caused by selective serotonin reuptake inhibitors (SSRIs)—such as oxidative stress, mitochondrial fission, and sarcomeric disorganization—can only be fully recapitulated in multi-cell organoids containing endothelial cells and appropriate stroma (Shen et al., 2025). These findings collectively demonstrate that models lacking multi-cellular interactions will systematically underestimate or misjudge drug toxicity.

In risk assessment for environmental and chemical toxicants, the combination of single-cell omics and biomanufacturing provides unique analytical capabilities. For example, a testing platform integrating bioengineered human liver and cardiac organoids generated quantitative multi-organ toxicity response profiles for toxins such as lead, mercury, thallium, and glyphosate through multi-parametric analysis (Forsythe et al., 2018). For microplastic exposure, hCO models not only exhibit oxidative stress, inflammation, and collagen deposition, but scRNA-seq further identifies the affected cell subpopulations and pathways (Zhou et al., 2023; Zhang et al., 2024); the inhibitory effect of low-dose bisphenol A on the hERG potassium channel and the resulting repolarization delay can only be fully captured in a perfusable microfluidic system, because shear stress significantly influences drug-channel interactions (Ma et al., 2023). The developmental cardiotoxicity of cadmium and glyphosate adjuvants also depends on the integrity of cell-matrix and cell-cell interactions within the three-dimensional multi-cellular environment (Wu et al., 2022; Sun et al., 2024). These cases illustrate that risk assessment of environmental toxicants must rely simultaneously on biomanufacturing and omics; otherwise, the actual risk will be severely underestimated.

In the field of cardiac regenerative therapy, strategies that fully integrate all three dimensions have shown promise for clinical translation. As previously mentioned, Voges et al. (Voges et al., 2017) have demonstrated that immature hCOs possess endogenous regenerative capacity under 3D perfusion and multicellular synergy. On this basis, Tan et al. (Tan et al., 2023) transplanted hCOs incorporating conductive nanowires (with an internal conductive network formed via biomanufacturing) into rat myocardial infarction areas, while preserving the multi-cellular structure comprising vascular endothelium and fibroblasts in the organoids, and verified the maturation of contractile proteins through proteomics. Compared to conditions without nanowires or injection of cell suspensions alone, nanowire-integrated hCOs significantly improved left ventricular ejection fraction and reduced adverse remodeling. Although still in the preclinical stage, the trial of engineered heart muscle allografts conducted by Jebran et al. (Jebran et al., 2025) in rhesus macaques and the first patient with heart failure has preliminarily demonstrated the feasibility, safety, and efficacy of the “structure–cell–molecule” three-level integration strategy in large animals and humans. Although the grafts used in that study were not strictly self-assembled hCOs, their design concept—spatial structure formed by bioprinting, vascularization supported by stromal cells, and quality control at the single-cell level is fully consistent with the integration framework described herein. Together, these advances suggest that future breakthroughs in cardiac regenerative therapy will depend on the synergistic application of all three dimensions, rather than the optimization of a single technology (Table 3).

**TABLE 3:** Emerging clinical transformation scenarios and values of hCOs.

Author	Year	Ref.	Evidence source/Study model	Key findings	Limitations
Voges HK, et al.	2017	Voges et al. (2017)	Regenerative Potential Study (Injury Model)	Developed a human cardiac organoid injury model, revealing its innate regenerative potential	Regenerative capacity limited to immature organoids; adult equivalence unknown; mechanism not elucidated
Mills RJ, et al.	2019	Mills et al. (2019)	Drug Screening Study (hPSC Cardiac Organoids)	Conducted drug screening in hPSC cardiac organoids and identified pro-proliferative compounds acting via the mevalonate pathway	Compound efficacy validated only *in vitro*; *in vivo* regenerative effect not tested; long-term safety unknown
Wu X, et al.	2025	Wu et al. (2025)	Disease/Toxicity Model (Doxorubicin)	Developed an advanced cardiac organoid model for studying doxorubicin-induced cardiotoxicity	No direct comparison with clinical patient data; ability to predict individual variability not validated
Harihara A, et al.	2025	Harihara et al. (2025)	Method Optimization Study	Evaluated the effects of seeding density on the health and functionality of cardiac organoids for toxicity studies	Only 1 cell line tested; multi-batch validation not performed
Jang J, et al.	2024	Jang et al. (2024)	Patient-Specific Disease Model (Breast Cancer/Doxorubicin)	Modeled doxorubicin-induced cardiotoxicity using breast cancer patient-specific iPSC-derived cardiac organoids	Only a few patient samples; genotype-phenotype association not deeply analyzed
Chen X, et al.	2023	Chen et al. (2023)	Toxicity Assessment Study (Doxorubicin)	Assessed doxorubicin toxicity using human cardiac organoids as a novel model for evaluating drug cardiotoxicity	Only a single drug concentration tested; no head-to-head comparison with traditional models
Beck TC, et al.	2022	Beck et al. (2022)	Mechanistic Study (MEK1 Inhibitor)	Revealed the cellular and molecular mechanisms of MEK1 inhibitor-induced cardiotoxicity	Mechanistic study relies mainly on pharmacological inhibition; other MEK inhibitors not tested
Shen Y, et al.	2025	Shen et al. (2025)	Developmental Toxicity/Mechanistic Study (SSRIs)	Selective serotonin reuptake inhibitors induce cardiac toxicity through dysfunction of mitochondria and sarcomeres	Drug concentrations may exceed clinical exposure levels; long-term developmental effects not assessed
Forsythe SD, et al.	2018	Forsythe et al. (2018)	Multi-Organ Toxicity Testing Platform	Used human-derived 3D bioengineered liver and cardiac organoids for environmental toxin screening	Only a limited number of toxins tested; insufficient crosstalk simulation between organs
Zhou Y, et al.	2023	Zhou et al. (2023)	Environmental Toxicant Study (Microplastics)	Low-dose polystyrene microplastics induce cardiotoxicity in mice and human-originated cardiac organoids	Microplastic exposure route differs from actual human ingestion
Zhang T, et al.	2024	Zhang et al. (2024)	Dynamic Toxicity Study (Nanoplastics)	Based on a cardiac organoid-on-a-chip, revealed dynamic insights into polystyrene nanoplastic-induced cardiotoxicity	Chip system complex, difficult to generalize; long-term low-dose effects not evaluated
Ma J, et al.	2023	Ma et al. (2023)	Toxicity Mechanism Study (Bisphenol A)	Low-dose bisphenol A and its analogs induce proarrhythmic toxicity in human cardiomyocytes and organoids by delaying cardiac repolarization	Mainly electrophysiological readouts, mechanical function not assessed; clinical relevance needs confirmation
Wu X, et al.	2022	Wu et al. (2022)	Developmental Toxicology Study (Cadmium)	Used human embryonic stem cells and cardiac organoids to study cardiac development in the presence of cadmium	Model simulates early development, not adult exposure; dose-response relationship needs refinement
Sun H, et al.	2024	Sun et al. (2024)	Developmental Toxicity Comparative Study (Herbicide Components)	Polyoxyethylene tallow amine and glyphosate exhibited different developmental toxicities in a human pluripotent stem cell-derived heart organoid model	Mixed exposure effects not assessed; underlying pathways not deeply dissected
Tan Y, et al.	2023	Tan et al. (2023)	Regenerative Therapy Strategy (Nanowired hCOs Transplantation)	Nanowired human cardiac organoid transplantation enables highly efficient and effective repair of infarcted hearts	Validated only in rats; long-term biosafety of nanomaterials not evaluated
Jebran AF, et al.	2025	Jebran et al. (2025)	Preclinical and Preliminary Clinical Study (Engineered Heart Muscle Graft)	Engineered heart muscle allografts for heart repair in primates and humans	Extremely small sample size;; long-term graft survival and functional maintenance require follow-up

## 5 Conclusions and future perspectives

As a product of interdisciplinary integration, the development of hCOs has benefited from technological breakthroughs across multiple fields, including stem cell technology, intelligent biomanufacturing, and single-cell omics. This review systematically outlines the innovative progress in hCOs from three dimensions: interdisciplinary technological innovation, disease mechanism research, and clinical translation applications. Compared to traditional reviews, this article highlights two core themes: “cross-disciplinary integration” and “clinical translation.” It focuses on elucidating innovative directions such as intelligent biomanufacturing, neuro-vascular-immune co-construction, and single-cell omics analysis, as well as emerging clinical translation scenarios like personalized drug screening, toxicity testing, and regenerative therapy. This provides a new perspective for research in this field.

Although each of the three technological dimensions has achieved breakthroughs on its own, their individual application suffers from inherent limitations. Intelligent biomanufacturing can generate organoids with controllable structures, but without precise molecular-level guidance, it may build upon an incomplete or incorrect cellular blueprint. Single-cell omics can produce high-resolution molecular maps, yet without functional validation in a perfusable 3D system, it remains descriptive. Multi-cell co-culture enhances physiological relevance, but blindly increasing cell types without a data-driven strategy leads to greater unpredictability and batch-to-batch variability. Overcoming these challenges relies on the synergistic integration of the three approaches: using single-cell omics to define quality-control indicators (e.g., cell type composition, maturation gene signatures) and adopting bioprinting to achieve standardized, reproducible organoid production; employing omics to identify key signaling pathways and then delivering mechanical or biochemical stimuli precisely via microfluidics to promote functional maturation; meanwhile, combining hiPSC technology, bioprinting, and single-cell omics enables both controllable construction and transcriptome-level validation in patient-specific models. This integration is not a simple superposition of technologies, but a closed loop from molecular blueprint to engineered construction to functional validation, the core innovation that distinguishes contemporary hCOs from earlier simplified models. For example, the three-dimensional microenvironment obtained through vascular endothelial-cardiomyocyte co-construction can faithfully recapitulate the effect of ET-1/PDGFRβ paracrine signaling on diastolic function; using a microfluidic perfusion system can simulate the dynamic oxygen gradient in ischemia-reperfusion and evaluate the efficacy of nanomedicines; combining single-cell transcriptomics allows validation of alterations in key molecular pathways in patient-specific organoids. In other words, the multidimensional integration strategy provides an indispensable experimental foundation for many discoveries, offering a reliable avenue for obtaining human-derived, multicellular, mechanosensitive mechanistic insights and predictive data.

In summary, hCOs represent a transformative bifunctional platform. They serve both as “patient-in-a-dish” models of genetic disorders, directly linking human genetics to pathophysiology, and as highly controlled phenotypic canvases for acquired diseases, enabling systematic dissection of environmental and pharmacological effects. Compared with traditional 2D cultures or animal models, hCOs have revealed novel mechanisms that were previously difficult to capture, as demonstrated in the multiple cases described above. Even when certain pathogenic mechanisms are conserved across different models, hCOs still enable new discoveries in a human context. For example, although the regulation of cardiomyocyte proliferation by the p38α/β and TGF-βR/BMPR pathways was already recognized, high-throughput functional screening using hCOs led researchers to further identify the mevalonate pathway as a previously unknown downstream component (Forsythe et al., 2018). These capabilities render hCOs uniquely advantageous for studying human-specific developmental or disease mechanisms when reliable animal models are lacking, for dissecting multicellular and mechanosensitive mechanisms that require three-dimensional architecture and mechanical signals, and for investigating personalized mechanisms that depend on patient genetic background.

Looking ahead, research in the field of hCOs is expected to follow three major development trends. First, interdisciplinary integration will deepen further. Advances in fields such as artificial intelligence (AI), synthetic biology, and nanotechnology will further empower the construction and application of hCOs. For instance, synthetic biology could be used to engineer stem cells to enhance the functional maturity of organoids, while nanotechnology may enable precise drug delivery to these models. Second, clinical translation scenarios will continue to expand. Beyond current applications in drug screening, toxicity testing, and regenerative therapy, hCOs may find future use in areas such as vaccine development for heart diseases and the evaluation of gene therapies. Third, the pace of technological standardization and industrialization will accelerate. With the establishment and refinement of industry standards, along with deeper collaboration between academia, research, and industry, hCOs are expected to progress toward scalable, automated production. Their economic accessibility is likely to improve significantly, holding potential for their adoption as routine clinical diagnostic and therapeutic tools.

However, we must also clearly recognize that the clinical translation of hCOs still faces many challenges: the functional maturity of organoids has not yet reached the level of native heart tissue, the stability of long-term culture needs to be improved, immune rejection remains a key bottleneck limiting transplantation applications, and ethical and regulatory frameworks still require further refinement. Furthermore, although hCOs have shown potential in drug screening, their predictive performance has not yet been systematically validated. There is currently a lack of statistical data on the clinical failure or withdrawal rates of candidate drugs evaluated using hCO-based platforms, as well as a lack of head-to-head drug response studies comparing hCOs with traditional models. In the future, multidisciplinary researchers must work together to overcome technical bottlenecks, improve ethical and regulatory systems, and promote the translation of hCOs from the laboratory to the clinic, which is expected to bring revolutionary breakthroughs in precision therapies for cardiovascular disease and advances in regenerative medicine.

## Glossary

ACTA1: Actin alpha 1, skeletal muscle
AI: Artificial intelligence
ANP: Atrial natriuretic peptide
ARVC: Arrhythmogenic right ventricular cardiomyopathy
BNP: Brain natriuretic peptide
BMPR: Bone morphogenetic protein receptor
BPA: Bisphenol A
CHD: Congenital heart disease
COL1A1: Collagen type I alpha 1 chain
CX43: Connexin 43
cTnT: Cardiac troponin T
DCM: Diabetic cardiomyopathy
DEC: Diabetic embryonic cardiomyopathy
DLK1: Delta-like 1 homolog
DMD: Duchenne muscular dystrophy
Drp1: Dynamin-related protein 1
ECM: Extracellular matrix
ET-1: Endothelin-1
FADS2: Fatty acid desaturase 2
FDA: Food and Drug Administration
GelMA: Gelatin methacryloyl
GelMA-Cad: N-cadherin peptide-functionalized gelatin methacryloyl
HAND1: Heart and neural crest derivatives expressed 1
HCM: Hypertrophic cardiomyopathy
hCOs: Human cardiac organoids
hiPSCs: Human induced pluripotent stem cells
IRE1: Inositol-requiring enzyme 1
PDGFRβ: Platelet-derived growth factor receptor beta
scRNA-seq: Single-cell RNA sequencing
SCN5A: Sodium voltage-gated channel alpha subunit 5
SSRIs: Selective serotonin reuptake inhibitors
sEVs: Small extracellular vesicles
TGF-βR: Transforming growth factor-beta receptor
VLCFAs: Very-long-chain fatty acids
WHF: World Heart Federation
2D: Two-dimensional
3D: Three-dimensional
ΔΨm: Mitochondrial membrane potential
